# Genome Sequence of the Versatile Deadwood Decomposer Xylaria grammica IHIA82

**DOI:** 10.1128/MRA.01306-20

**Published:** 2021-01-07

**Authors:** Enrico Büttner, Virginia Wambui Kimani, Harald Kellner, Martin Hofrichter, Christiane Liers

**Affiliations:** a Department of Bio- and Environmental Sciences, International Institute Zittau, Technische Universität Dresden, Zittau, Germany; Broad Institute

## Abstract

Xylaria grammica is an ascomycetous decomposer of dead hardwood. The *X. grammica* strain IHIA82 was recovered from the Kakamega Forest in Kenya. The whole genome of this strain was sequenced with a total size of 47.0 Mbp, a G+C content of 48.1%, and 12,126 predicted genes.

## Abstract (precis)

Xylaria grammica is an ascomycetous decomposer of dead hardwood. *X. grammica* IHIA82 was recovered from the Kakamega Forest, Kenya. The whole genome of this strain was sequenced with a total size of 47.0 Mbp, a G+C content of 48.1%, and 12,126 predicted genes.

## ANNOUNCEMENT

Xylaria grammica (Mont.) Mont. 1855 belongs to the ascomycete family Xylariaceae and is related to Xylaria hypoxylon (1, 2). The fungus is predominantly found in tropical Africa, America, Asia, and Australia, where it grows on deciduous wood. Besides its interesting spectrum of secondary metabolites (e.g., grammicin, a nematicide [3, 4]), *X. grammica* causes soft-rot type II and is therefore a suitable candidate to examine for extracellular enzymes with promising biotechnological potential. The species sequenced here was collected in tropical Africa; thus, this research contributes to a better understanding of the so-far-underexplored fungal biodiversity of this continent.

*Xylaria grammica* IHIA82 (ribosomal cistron, GenBank accession number MK408621; proteins RPB2, β-tubulin, and Tef1α, RWA13214, RWA14836, and RWA10218, respectively) was collected from rotting plant debris in the Kakamega Forest National Reserve (Kenya; lat 0.33431, long 34.87814). Mycelium was grown in agitated liquid culture (2.5% malt medium), and genomic DNA was extracted using a standard cetyltrimethylammonium bromide (CTAB)-based protocol. The purified DNA was sheared into 200-bp fragments using a Covaris S2 sonicator (Woodingdean, Brighton, UK). A 200-bp fragment library (Ion Xpress Plus fragment library kit) was subsequently generated and sequenced using the Ion Torrent Personal Genome Machine (PGM) platform (Ion PGM sequencing 200 kit v2, 318v2 chip, Thermo Fisher, Darmstadt, Germany). Altogether, 5.5 million quality-filtered sequence reads were trimmed using Geneious Prime v2019.2 (length, >180 bp; error probability limit, 0.05; trim 3′ end) (5). *De novo* assembly was performed using MIRA v4.0 (minimum reads per contig, 100 [6]), and in a second step, a Geneious assembler (highest sensitivity [5]) was used to join the contig ends and to filter for duplicate contigs. Assembly resulted in 1,053 contigs (969 chromosomal and 84 mitochondrial contigs) with a total size of 47.0 Mbp and a G+C content of 48.1%; the largest contig comprised 494,172 bp. Assembly quality (coverage, 29.6×) was assessed using QUAST v4.5 (7) and resulted in *N*_50_ and *L*_50_ values of 82,670 bp and 172, respectively. Single-copy ortholog analysis performed with BUSCO v3 (predictor, Aspergillus nidulans; fungal data set, Ascomycota_*odb9*) (8) reported a genome completeness of 93.7%. Gene prediction was performed using the AUGUSTUS v3.2.2 Web server (predictor, A. nidulans) (9) and resulted in 12,126 protein-coding genes. Genes were annotated using Blast2GO v5.2.5 (BioBam, Valencia, Spain) and dbCAN (HMMdb v7; E value, <1e^−15^; coverage, >0.35×) (10). Altogether, 753 carbohydrate-active enzymes (CAZys; among them, 295 glycoside hydrolases and 165 enzymes with auxiliary activities) and related binding modules were identified. Oxidative enzymes involved in lignocellulose decomposition and the conversion of aromatics such as lytic polysaccharide monooxygenases, cellobiose dehydrogenases, dye-decolorizing peroxidases, and heme-thiolate peroxidases were identified by BLAST searches and annotated manually and are available under the GenPept accession numbers listed in Table 1. Secondary metabolite (SM) biosynthetic gene clusters (BGCs) were predicted using antiSMASH v4.1.0 (11). We identified 47 BGCs, including BGCs for the synthesis of 31 type 1 polyketides, 11 nonribosomal peptides, and 10 terpenes. A more detailed analysis of SM anchor genes, e.g., polyketide synthase (PKS), nonribosomal peptide synthetase (NRPS), and dimethylallyl tryptophan synthases (DMATS), was performed using Secondary Metabolites by InterProScan (SMIPS v3 [12]) and is summarized in Table 1.

**TABLE 1:** CAZy classes, SMIPS, and antiSMASH identification for (anchor) genes, secondary metabolite types, and clusters in the genome sequence of *X. grammica* IHIA82

Particle group and type^*a*^	No. of proteins	GenPept accession no.
CAZy classes		
Glycoside hydrolase	295	
Glycosyltransferase	97	
Polysaccharide lyase	18	
Carbohydrate esterase	117	
Auxiliary activities	165	
Associated modules		
Carbohydrate-binding module	61	
Cellulose-binding domain CBM1	12	
Oxidoreductases		
Unspecific peroxygenase	5	RWA12854.1, RWA12535.1, RWA09762.1, RWA08467.1, RWA07285.1
Dye-decolorizing peroxidase	3	RWA14623.1, RWA13170.1, RWA05922.1
Lytic polysaccharide monooxygenase	23	RWA05857.1, RWA07035.1, RWA14554.1, RWA09097.1, RWA12945.1, RWA08580.1, RWA14079.1, RWA13280.1, RWA10304.1, RWA10363.1, RWA05537.1, RWA09241.1, RWA04958.1, RWA13290.1, RWA11494.1, RWA11080.1, RWA14855.1, RWA10346.1, RWA12163.1, RWA12842.1, RWA12711.1, RWA03274.1, RWA06405.1
Cellobiose dehydrogenase	2	RWA13597.1, RWA11079.1
Secondary metabolites	
NRPS genes	62
DMATS	13
NRPS	11
NRPS-PKS hybrid	7
PKS	31
NRPS- and PKS-like genes^*b*^	10/23^*c*^
NRPS-like	0/18^*c*^
PKS-like	10/5^*c*^
Single-domain genes	25
AT	20
KS	5
Cluster genes	11
Terpene synthase	10
Fungal-RiPP	1

a NRPS, nonribosomal peptide synthetase; DMATS, dimethylallyl tryptophan synthase; PKS, polyketide synthase; AT, acyl transferase; KS, beta-ketoacyl synthase; RiPP, ribosomally synthesized and post-translationally modified peptide.

b Incomplete anchor genes, one KS and/or C domain.

c Incomplete anchor genes, with two typical PKS and/or NRPS domains.

### Data availability.

This whole-genome shotgun sequencing project was deposited at DDBJ/ENA/GenBank under accession number RYZI00000000. The version described here is the first version, RYZI01000000. The Sequence Read Archive (SRA) accession number is SRR8352207. All referenced genes are cited within BioProject number PRJNA510724.
